# Vesicular Nanocarriers for Phytocompounds in Wound Care: Preparation and Characterization

**DOI:** 10.3390/pharmaceutics14050991

**Published:** 2022-05-05

**Authors:** Diana Antonia Safta, Cătălina Bogdan, Mirela Liliana Moldovan

**Affiliations:** Department of Dermopharmacy and Cosmetics, Faculty of Pharmacy, “Iuliu Hațieganu” University of Medicine and Pharmacy, 12 I. Creanga Street, 400010 Cluj-Napoca, Romania; diana.an.safta@elearn.umfcluj.ro (D.A.S.); mmoldovan@umfcluj.ro (M.L.M.)

**Keywords:** herbal extracts, phytocompounds, lipid vesicles, vesicular nanosystems, nanotechnology, wound healing, vesicles’ preparation, vesicles’ characterization

## Abstract

The need to develop wound healing preparations is a pressing challenge given the limitations of the current treatment and the rising prevalence of impaired healing wounds. Although herbal extracts have been used for many years to treat skin disorders, due to their wound healing, anti-inflammatory, antimicrobial, and antioxidant effects, their efficacy can be questionable because of their poor bioavailability and stability issues. Nanotechnology offers an opportunity to revolutionize wound healing therapies by including herbal compounds in nanosystems. Particularly, vesicular nanosystems exhibit beneficial properties, such as biocompatibility, targeted and sustained delivery capacity, and increased phytocompounds’ bioavailability and protection, conferring them a great potential for future applications in wound care. This review summarizes the beneficial effects of phytocompounds in wound healing and emphasizes the advantages of their entrapment in vesicular nanosystems. Different types of lipid nanocarriers are presented (liposomes, niosomes, transferosomes, ethosomes, cubosomes, and their derivates’ systems), highlighting their applications as carriers for phytocompounds in wound care, with the presentation of the state-of-art in this field. The methods of preparation, characterization, and evaluation are also described, underlining the properties that ensure good in vitro and in vivo performance. Finally, future directions of topical systems in which vesicle-bearing herbal extracts or phytocompounds can be incorporated are pointed out, as their development is emerging as a promising strategy.

## 1. Introduction

Wound healing is a complex process with great importance as healthy skin is a key to the maintenance of body homeostasis. The increasing prevalence of acute, surgical, and chronic wounds is expected to raise the global costs of wound care, up to USD 18.7 billion by 2027, and an increasing percentage of the health sector budget is assigned to chronic wound care. Expensive treatments, wound chronicity, and poor healing leading to long-term hospitalization represent the substantial challenges of actual wound-care products. There is a growing need for wound management products to control complex wounds, in the context of the increase of the aging population and the growing prevalence of diseases and conditions affecting the wound healing process, such as diabetes and obesity. Additionally, wound infection represents a major obstacle in the healing process, and the widespread bacterial resistance may contribute to increasing the rate of acute and chronic wounds. It is considered that 25% of diabetic patients will develop foot ulcers during their lifetime and about 60% of these patients will develop infections and subsequent complications [1,2]. Scleroderma skin ulcers, a consequence of systemic sclerosis, represent another category of skin lesions for which local therapy, such as chronic wound care, is critical [3,4].

The conventional strategies do not fully cover the large variety of wounds and the growing need for healing products. The significant costs for healthcare systems and the insufficient control of complex wounds underline the limited effectiveness of the current approaches. Although remarkable progress in wound healing research was recorded in the last one hundred years, gaps in wound research still exist [5]. Therefore, the development of new topical systems for the treatment of wounds and healing without scars or other complications is an intensely studied field nowadays.

Currently, research in wound healing is focusing on the development of vesicular systems with enhanced delivery performances [6]. Particularly, in the past years, special efforts have been made in the development of new, safe, green, scalable, and affordable delivery systems capable of transforming traditional herbal medicines into effective health-promoting products [7].

Herbal extracts (HEs) and phytocompounds (PCs) are the subject of considerable interest for their complex beneficial effects on skin health, such as regenerative, anti-inflammatory, antioxidant, and antimicrobial effects. During the last decade, among other versatile agents studied for wound healing, the plant-derived ingredients, also called phyto-drugs, have attracted great attention because they are safe, easily available, and affordable [8,9,10,11]. Despite their beneficial effects in wound healing, their bioavailability is reduced because of poor membrane permeability and instability issues. Therefore, novel drug-delivery strategies including the incorporation of these PCs into delivery systems such as vesicular nanosystems (VNs) have been developed to overcome these limitations [12]. To date, a considerable number of studies have been conducted to develop different types of vesicles, such as liposomes, niosomes, ethosomes, transferosomes, cubosomes, and their derivates, but also to optimize the characteristics of these lipid vesicles.

In the context of the increasing rate of acute and chronic wounds, this topic is more relevant than ever. The development of novel delivery platforms based on PCs requires a deep knowledge of the current evidence. Thus, the current review article intends to support the scientists to find updated information by gathering recent relevant research undertaken so far in this field and contributes to a thorough understanding of the materials’ selection and preparation method. The advances in the field are discussed, emphasizing the advantages of the entrapment of HEs and PCs in different types of lipid nanovesicles for the treatment of wounds, together with their methods of preparation and characterization. The main features concerning the use of PCs in wound care, but also the challenges associated with the incorporation of vesicular carriers into wound dressings, are discussed.

## 2. Presentation of the Cellular and Molecular Mechanisms of Wound Healing

The skin is the largest organ of the body and fulfills essential roles such as protection, thermoregulation, aesthetics, and sensory perception. Therefore, the integrity and functionality of the skin are critical. If the tissue suffers a lesion of various etiologies, the dynamic and complex physiological healing process is triggered [13]. It involves four successive and partially overlapping stages: hemostasis, the inflammatory phase, the proliferative phase, or granulation and maturation, also called remodeling. During these stages, numerous changes and interactions take place between the cells and the mediators at the wound site, leading to the healing of the tissue, with the restoration of the integrity and functions of the skin [5,14,15].

**Figure 1:**
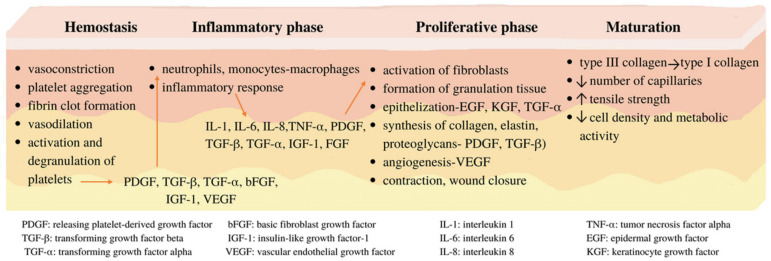
The main stages of the wound-healing process.

The first stage of healing, hemostasis, aims to stop the bleeding caused by tissue damage, with vasoconstriction, platelet aggregation, and fibrin clot formation, which lead to cessation of bleeding by coagulation cascade. Further, blood vessels dilate to allow essential cells to reach the site of the injury. Platelets activate and degranulate, releasing platelet-derived growth factor (PDGF), transforming growth factor beta (TGF-β), transforming growth factor alpha (TGF-α), basic fibroblast growth factor (bFGF), insulin-like growth factor-1 (IGF-1), and vascular endothelial growth factor (VEGF). Following the cytokines’ release, neutrophils and monocytes-macrophages are recruited to initiate the inflammatory response. They prevent the infection, cleanse the wound of debris, and release soluble mediators such as proinflammatory cytokines (interleukins: IL-1, IL-6, IL-8, and tumor necrosis factor alpha: TNF-α) and growth factors (such as PDGF, TGF-β, TGF-α, IGF-1, and FGF) that are involved in the recruitment and activation of fibroblasts and epithelial cells, making the transition to the next phase in healing. The proliferative phase consists of the granulation tissue formation, epithelialization by means of epidermal growth factor (EGF), keratinocyte growth factor (KGF), and TGF-α, followed by fibroblast migration and extracellular matrix production by synthesis of collagen, elastin, proteoglycans in fibroblasts (employing PDGF and TGF-β), angiogenesis (formation of new blood vessels by VEGF), and contraction, ending with wound closure. Maturation or remodeling of the lesion is the last stage of the healing process, during which structural reorganizations occur. Thus, type III collagen, initially synthesized at high levels, is replaced by type I collagen, the dominant fibrillar collagen in the skin. Maturation also implies the reduction in the number of capillaries, increase in tensile strength, and decrease in cell density and metabolic activity in the granulation tissue [5,14,15,16,17]. A summarization of the wound-healing process is presented in Figure 1.

Often, the physiological healing process is not effective and fast enough, and therefore specific topical systems help patients to achieve optimal healing, by preventing infections and complications, and ensuring skin regeneration with minimal scarring [18]. The use of healing products is important, especially for chronic wounds, with poor healing, such as varicose veins, pressure ulcers or diabetic foot ulcers, bedsores, but also for healing acute wounds caused by abrasions, lacerations, cuts, surgery, or burns, which affect the patient’s quality of life. They also predispose to complications, such as defective scarring, infections, and their extensions, which can even lead to bone infections, necrosis, amputations, etc. In addition, the high incidence of these diseases leads to increased costs in the medical system and the imperious need for efficient and economically advantageous products for the treatment of wounds [9]. Therefore, numerous studies related to the healing process are currently underway, with many of them aiming to understand the targets by which scarring agents can act. Elucidating the complex mechanisms of wound healing supports the development of products that improve this process, leading to rapid and effective wound healing.

## 3. The Role of the Phytocompounds in Wound Care

The importance of natural PCs in all health disorders is well-known. Some of the PCs stand out for their effectiveness in the treatment of wounds because they stimulate the growth factors involved in the process of healing, regulate collagen synthesis, and promote its deposition and the closure of the wound. Briefly, they can be wound-healing agents. Among them, *Aloe vera* [19] was intensely studied for its wound-healing properties. The whole extract, rich in acemannan and β-sitosterol, can stimulate the expression of VEGF and potentiates the synthesis of nitric oxide (NO) by regulating NO synthase (NOS) activity [19]. Acemannan from *Aloe vera* is a polysaccharide able to increase fibroblast proliferation, the release of KGF-1 and VEGF, and the synthesis of type I collagen, being an important agent for tissue re-epithelization and angiogenesis [20]. Another well-known plant with wound healing properties is *Hypericum perforatum* [21,22], which enhances the migration of fibroblasts, collagen deposition, and by the action of hyperforin, PC, that is also antibacterial, increases the re-epithelization [21,22].

Plant-derived antioxidants may counteract the damaging effects of oxidative species in human tissues and can also accelerate the healing of wounds by regulating the redox environment [7,23]. It is well-known that skin lesions are usually associated with an important level of oxidative species [7]. Firstly, the reactive oxygen species (ROS) have a key role as mediators of intracellular signaling involved in the defense against the acquired pathogens in dermal injuries. On the other hand, excessive amounts of ROS are harmful because of the generation of free radicals and the oxidation of cellular components. Insufficient scavenging of the excessive amounts of ROS can affect the wound-healing process, resulting in delayed healing, an impaired or chronic wound, and even neoplastic transformation [23,24]. The topical application of compounds with free-radical scavenging and anti-inflammatory properties, such as quercetin, curcumin, and other polyphenols, has shown a significant improvement in wound healing and protection from oxidative damage [25]. Polyphenolic compounds can scavenge the ROS released during the inflammatory process, thus attenuating inflammation, and also having antioxidant and anti-inflammatory properties [26,27].

An important biological property of HEs, which is relevant in wound care, is represented by the anti-inflammatory effect. In this sense, terpenoids have strong antioxidant and anti-inflammatory as well as antimicrobial properties. However, the pharmaceutical applications of terpenoids remain challenging due to their poor water solubility and high volatility [28]. The aqueous extract of *Moringa oleifera* [29] was also found to reduce the levels of inflammatory markers in macrophages, including induced nitric oxide synthase (iNOS), TNF-α, and IL-1β [29]. Another PC with an anti-inflammatory effect is glycyrrhizin, from *Glycyrrhiza glabra* [30] extract, which inhibits inflammatory events, such as edema, apoptosis, iNOS expression, and NF-kB [30]. A less-studied PC with beneficial effects in wound healing, as an anti-inflammatory and antibacterial agent, is usnic acid, extracted from different lichens species [31]. According to a study conducted by Zhang et al., application of the sodium salt of usnic acid triggered the complete re-epithelialization, formation of well-organized bands of collagen, and epidermal keratinization. These effects are due to its capacity to decrease the inflammatory cells and to increase fibroblast proliferation, granulation tissue, and vascular regeneration by increasing VEGF levels [32].

Another important property of HEs or PCs used in the treatment of wounds is represented by the antibacterial activity. They should be active on the bacteria most commonly found in wound infections, such as aerobic bacteria—*Staphylococcus aureus* and resistant strains (e.g., MRSA: methicillin-resistant *Staphylococcus aureus*), some species of *Enterococcus* (the most abundant in diabetic wounds, in immune-compromised patients and abdominal surgeries), facultatively anaerobic bacteria, such as *Escherichia coli* and *Proteus mirabilis* frequently met in chronic wounds, as well as anaerobes, such as *Pseudomonas aeruginosa* (commonly isolated from wounds following surgeries and burns) [33]. Another risk factor for poor wound healing may be the infection with fungal species, of which *Candida* spp. can most often colonize burn wounds [34,35]. In this regard, essential oils from *Thymus* sp., *Citrus* sp., or terpenes isolated from oils are known for their antibacterial activity on strains involved in skin infection [36,37].

**Figure 2:**
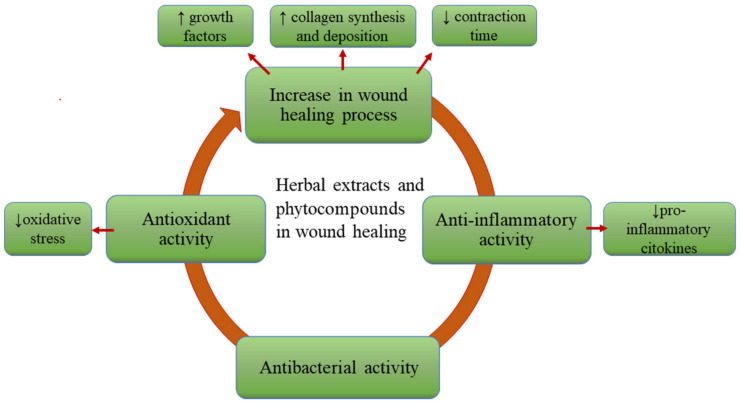
The main beneficial effects of herbal extracts and phytocompounds in wound care.

Recent studies are reported to elucidate the wound healing mechanisms of PCs. Although they have been used empirically for centuries in the treatment of various affections, including skin disorders, lately, relevant research has been undertaken to validate their biological activity. Furthermore, their entrapment into topical systems to ensure the best effect is challenging [8,9,38,39]. The main beneficial effects of HEs in wound healing are presented in Figure 2.

## 4. The Entrapment of Herbal Extracts into Vesicular Nanosystems—Challenges in the Formulation

A modern approach for improving the efficacy of phytocompounds at the skin level is represented by their incorporation into nanocarriers [40]. Among the high number of nanocarriers, phospholipid vesicles represent one of the most valuable and versatile systems, especially for skin delivery, owing to their structure, biocompatibility, and similarity to skin components [7,41]. Moreover, VNs have multiple advantages of enhancing the stability of loaded substances, preventing physical and chemical degradation induced by light, air, acid, and alkali, increasing the bioavailability of PCs, and improving their penetration across the skin. These advantages are ascribed to their size, elasticity, and lipid content, which facilitate the interaction with the skin layers. Moreover, phospholipid vesicles are biocompatible and non-toxic nanosystems, being able to incorporate and deliver PC molecules at the desired site of action [42,43,44]. The improvement of the effectiveness of molecules entrapped into phospholipid vesicles and accumulation into deep skin layers when applied topically is demonstrated by many studies. So far, the results provided by the experimental research revealed that each HE or PC requires a specific ad hoc tailored phospholipid vesicle formulation to maximize its efficacy [45,46].

To be effective in wound healing, the PCs must penetrate the skin to a significant extent. The absorption into the skin of topically applied molecules, including PCs, is governed by their partition coefficient, a function of their lipophilicity. Among the routes of skin penetration, the transepidermal route is important in this case. Hydrophilic molecules can pass through the transcellular route as an aqueous hydrophilic pathway, through the cytoplasm of keratinocytes and phospholipidic membranes. Although this is the shortest route, it encounters significant permeation resistance because the molecules must pass via the lipophilic membrane of each cell, being subjected to multiple partitioning and diffusion steps. Active molecules can also pass through the continuous lipid matrix and the small spaces between cells, via the intercellular pathway. Large molecules, such as PCs, are physically bound within the lipids for passing through the skin via an intercellular route, being known as the suitable absorption route for most penetrants [47]. The transfollicular route has also recently been identified as a significant penetration pathway. Nanoparticles, in particular, have a higher intrafollicular penetration rate and can be used to target specific cell populations within the hair follicle [48]. Liposomes penetrate as an intracellular delivery system and deposit the entrapped substances in the stratum corneum, having low drug penetration into the dermis. To release the medication into the dermis, transferosomes squeeze through the intercellular sections of the corneous layer or penetrate through the transfollicular pathway. Ethosomes use fluidization to permeate lipids in the stratum corneum, then release the actives into the dermis through transfollicular or intercellular routes [16]. Moreover, a few studies have assessed absorption through damaged human skin in vitro, showing a modest but clear increase in absorption compared to intact skin, more favorable for hydrophilic molecules [49].

A major impediment in percutaneous absorption is represented by the polar chemical groups from the structure of actives of herbal origin, such as hydroxyl substitutes and glycosides. Besides this, the natural compounds have large molecular weight and poor membrane permeability [44]. Most of the biologically active PCs, mainly the flavonoids, phenolics, and glycosidic aglycones, have limited bioavailability in the cellular media [50]. To solve this problem, the encapsulation in VNs seems to bring benefits, because in this way, the drug penetration into cell membranes is facilitated, increasing the permeability of active substances and topical therapeutic effectiveness [21,29]. The choice of the topical system has a decisive role concerning the penetration into the epidermis and maintenance of the therapeutic concentration in the skin tissue for a determined period [44,48]. It has been reported that liposomal formulations provide sufficient penetration of these molecules to the target tissue while minimizing systemic side effects and prolonging local therapeutic action. Additionally, the VNs are considered promising delivery systems due to the ability of the phospholipidic structure to mimic the structural component of the mammalian cell membrane. Thus, the active compounds can passively penetrate the lipophilic membrane without causing cell damage [21,29].

Another major obstacle in the utilization of phenolic antioxidant compounds is represented by their poor stability. Therefore, they are prone to degradation by the influence of various factors, such as high oxygen levels or alkaline pH. These compounds can be entrapped into a carrier system to protect them against degradation and oxidation, to increase the stability of these molecules during storage, and to maintain their antioxidant activity, important for the healing of the wound. Due to the presence of phospholipidic vesicle membranes, the reaction between oxygen and polyphenols is limited. Additionally, due to the slow release from the carrier system of the entrapped polyphenols, their degradation is less important in comparison to free polyphenols [51].

Essential oils, which are known for their antibacterial activity, can be effective in infected wound healing, but they have low stability due to their volatility [36]. Likewise, the essential oils’ encapsulation in nanosystems can reduce their volatility and chemical instability, without changing their chemical ingredients, and can increase their safety. By encapsulation, the antimicrobial activity could be improved, while preserving or even increasing their clinical effectiveness. This is mainly due to the subcellular size of the VNs, which could strengthen the passive absorption mechanism and reduce the transport resistance of the substances [52,53].

**Figure 3:**
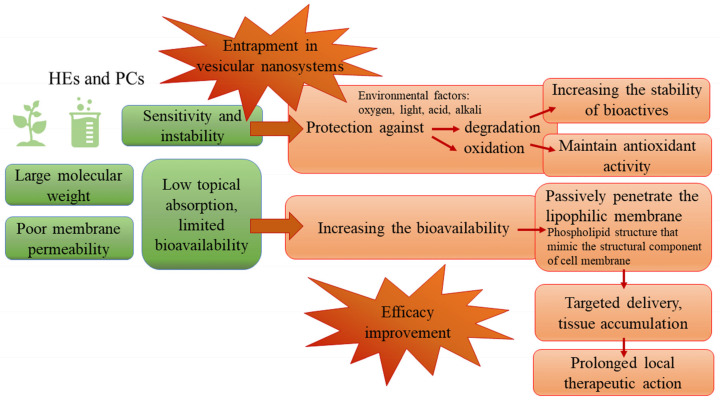
Advantages of herbal extracts’ entrapment into vesicular nanosystems.

Among the lipid-based entrapment technologies, liposomes have been the most successfully applied systems due to their numerous advantages, including the use of natural raw materials, the ability to entrap PCs with different solubilities, and the prevention of ingredient oxidation by free radicals, metal ions, and enzymes [51]. The property of liposomes to improve the cutaneous delivery of molecules with various molecular sizes is well-known. Recently, they have been successfully proposed for the skin delivery of PCs due to the increased loading efficiency and carrier abilities, as well as the affinity for different skin layers [36]. Therefore, phyto-phospholipidic complex formulations are exhaustively explored [21,29]. Figure 3 summarizes the main advantages of the entrapment of PCs in VNs and highlights the overcoming of the bioavailability issues through the encapsulation in phospholipid vesicles.

Besides the advantages of using vesicular nanosystems for wound care, the evaluation of safety for human health and the environment is very important in the development of nanomaterials. Thus, when evaluating novel topical herbal nanoformulations, a thorough assessment of safety is essential, taking into account issues such as toxicity, which can be attributed to either the nanosystem components or the herbal drug itself [54]. To this aim, a suitable characterization of the nanosystems should be performed [55]. Taking into consideration that in general the components of vesicular nanocarriers of HEs and PCs are biocompatible, environmental safety should not be a concern. Even after topical application, besides poor dermal penetration which limits their potential toxic effects, there is a probability of nanoparticles attaining systemic circulation, where they may interact unexpectedly with immune system cells or produce free radicals [54,55]. Thus, toxicity testing of products is necessary to ensure their safety [56].

## 5. Types of Vesicular Nanosystems

### 5.1. Liposomes

Liposomes are considered one of the most versatile nanocarrier systems [36], represented by colloidal systems, usually consisting of a central aqueous compartment (or more) surrounded by one or more concentric phospholipid bilayers, produced by self-assembly [51,57]. Hydrophilic substances can be encapsulated in the interior aqueous compartment, lipophilic drugs within lipid bilayers, and amphiphilic molecules can also be included in vesicles at the lipid/water interface. Liposomes are intensively studied and well-recognized as pharmaceutical carriers for the reduction of toxicity of encapsulated substances and efficacy enhancement, being able to provide and enhance the passage of active compounds in the skin due to the similarity to cell membrane structure [57,58]. They are biocompatible, biodegradable, non-immunogenic, and non-toxic systems widely studied for topical applications [57,58], recognized as the safest and most suitable nanosystems for application in human patients with high compatibility due to the membrane composition, phospholipids, and cholesterol, identical to cells’ components [59].

Studies have shown that the antioxidant and immunomodulatory properties of lecithin liposomes might be effective in the improvement of wound healing as well as dermal drug-delivery systems. A study conducted by Nasab et al. compared the antioxidative and wound-healing effects of egg lecithin and soy lecithin liposomes, highlighting the increasing rate of radical scavenging activity of the liposomal lecithins. Wound-healing assessments showed a significant effect in the treatment with topical lecithin liposomes and better outcomes in the excision wound model of egg-lecithin in comparison to phenytoin 1% cream [23]. However, the incorporation of poorly water-soluble drugs in lipid bilayers is often limited, and the hydrophobic molecules can be rapidly released from the lipid bilayers. This may limit the potential application of liposome carriers in the case of hydrophobic molecules [57]. In addition, traditional liposomes have a limited penetration ability, since they cannot penetrate the stratum corneum and can only deliver drugs to the surface of the skin, without achieving the effect of deep penetration [60,61]. Another disadvantage of traditional liposomes is the peculiar rigidity of the lipid bilayer, which limits their application as topical drugs. On the contrary, ethosomes, and transferosomes, newer liposomal systems, are characterized by adequate deformability, which allows the interaction with skin structures and passage through the stratum corneum [62]. A comparative study between curcumin liposomes and nanoplexes, a novel drug nanoparticle complex with the oppositely charged polyelectrolyte, showed that curcumin liposomes were more efficient for scar treatment and exhibited faster in vivo wound healing than nanoplexes [59].

Though the structural membrane components, lipids, are biocompatible, their long-term stability is limited [21]. Taking this into consideration, novel carriers, namely polymersomes or hybrid lipid/polymer vesicles, have been developed. In this way, the inherent advantages of their components, such as the biocompatibility of lipids and the mechanical stability, were combined with the chemical versatility of copolymers [63,64]. These vesicles are composed of amphiphilic block copolymers, including polydimethylsiloxane, polybutadiene, and polyisobutylene, which are extensively investigated [65]. In comparison to liposomes, polymersomes exhibit superior stability, but insufficient permeability, which may hamper the release of the encapsulated substances. To solve this problem, many methods, such as the design of stimuli-responsive polymersomes, the introduction of channel proteins within bilayers, and the post-modification of bilayer membranes, have been proposed to regulate their permeability [63,64]. Stimulus-controlled drug delivery from polymersomes has been demonstrated as well, using triggers such as pH or temperature changes [65]. The interest of the scientific community in these polymeric vesicles has risen, but the research on this topic is still in its early stages. Regarding the topical systems for wound healing, only a few studies describe the development of polymersomes, such as antibacterial polymersome-based wound dressing spray with cysteine [66], polymersomes with nitric oxide for corneal wound healing [64], and polymersomes with trophic factors [65].

Invasomes are liposomes composed of unsaturated phospholipids, such as phosphatidylcholine, small amounts of ethanol, one or more different terpenes, and water. In addition to biological activity due to the antimicrobial, anti-inflammatory, and strong antioxidant properties, terpenoids can also serve as permeation enhancers for transdermal delivery and bioavailability enhancers [28]. Recent research shows that terpenes may enhance skin permeation through disruption of the stratum corneum lipids, interacting with intracellular proteins, and improving the distribution of the drug into the stratum corneum. Ethanol also improves the ability of the vesicles to penetrate the stratum corneum and provides a net negative surface charge, preventing vesicle aggregation due to electrostatic repulsion. Thus, it is underlined that invasomes enhance percutaneous penetration compared to conventional liposomes, due to a synergistic effect between terpenes and ethanol, showing promising results in increasing the bioavailability of terpenoid-based drugs, that can be further applied for treating severe bacterial infections [28]. An example of the skin application of invasomes is the entrapment of dapsone intended for the treatment of acne [67].

The study conducted by Castangia et al. described the development of new liposome-derived vesicles called santosomes, formulated using *Santolina insularis* essential oil [60], phosphatidylcholine, and propylene glycol. Santosomes were loaded with phycocyanin, a natural phycobiliprotein of blue-green algae with wound-healing properties. The essential oil of *Santolina insularis* played a double role in modifying the bilayer structure and, on the other hand, in improving the delivery of the loaded drug due to a synergistic effect of the terpenes and phospholipids [60].

Phytosomes or herbosomes are vesicular systems obtained through the interaction between hydrophilic parts of the phospholipids and the phyto-active components, resulting in the formation of hydrogen bonds between them [19,29,68]. Phytosomes are considered a type of liposomal formulation, also called phyto-phospholipid complexes. The structural difference between phytosomes and liposomes is that phytosomes entrap the vegetal active ingredient as a part of the phospholipidic membrane itself, bound in their structure, while liposomes have their active ingredient inside the hydrophilic cavity or within the layers of membranes. More specifically, the synthesis of phytosomes is based on the stoichiometric reaction between phosphatidylcholine (polar heads of phospholipids, e.g., positive ammonium and negative phosphate groups) and polyphenolic constituents or standardized extracts (flavonoids, tannins, terpenoids, xanthones) in an aprotic solvent. Of all the phytocompounds, only those having an active hydrogen atom (e.g., -COOH, -OH, -NH_2_, -NH), such as polyphenols, can be integrated into a phytosome structure by forming hydrogen bonds between the herbal derivatives and the hydrophilic parts of amphiphilic molecules [69].

The main beneficial properties of phytosomes include high carrier loading capacity of substances, stability, ease of storage in a solid ready-to-reconstruct state, small size, and biocompatibility, making them suitable carriers for highly water-soluble drugs [19]. Other advantages are represented by the increase of the absorption rate of lipid insoluble phytomaterials, leading to more stable formulations within the formation of chemical bonds but also to an improved therapeutic benefit while decreasing the required dose for attaining the effect [70]. Alongside this, achieving a nano-sized phytosomal formulation or nanophytosome can further improve the bioavailability and distribution [19]. Since phytosomes exhibit a chemical bonding between phospholipid molecules and PCs, they are known for their improved stability [71].

A phytosomal formulation with *Aloe vera* and l-carnosine extract intended for type II diabetes mellitus-associated microvascular complications with an impaired angiogenesis background was developed by Darvishi et al., revealing a wound-healing effect of the formulation [19]. AuNP (gold nanoparticle) phytosomes with *Calendula officinalis* [70] extract have been prepared and characterized by Demir et al., with antioxidant and wound-healing effects of the product being reported [70]. Another phytosomal formulation with wound-healing effects was developed by Lim et al., by entrapment of *Moringa oleifera* extract [29].

Glycerosomes represent a novel type of modified liposomes containing glycerol, as a penetration enhancer. These vesicles are exploited to deliver PCs, such as extracts or oils, leading to an enhanced skin bioavailability of the encapsulated PCs, in comparison to the classical liposomes [37,45]. It is emphasized that glycerosomes can promote the accumulation of PC molecules in the different skin layers, mainly because of the moisturizing effect of glycerol, which may modify the ordered structure of the stratum corneum and favor the passage of the active substances [45]. Glycerosomes can be prepared by replacing a certain amount of water included in the composition of liposomes with glycerol [26].

There are several studies in which HEs or the phytocomponents were included in glycerosomes or derivate vesicles for the treatment of wounds. In a study undertaken by Allaw et al., glycerosomes including *Hypericum scruglii* [45] extract were modified through the addition of maltodextrin (glucidex) and a polymer (gelatin or hyaluronan) to obtain gluglycerosomes and gel-gluglycerosomes. The study aimed to improve the performance of the vesicles by increasing the stability and the viscosity of the dispersions and by enhancing their ability to deliver the payloads to the wound site [45]. In another study conducted by Manconi et al., gly-hyalurosomes with *Citrus limon* var. *pompia* [26] extract have been developed by adding sodium hyaluronate to liposomes and glycerosomes, to prevent the oxidative damage and death of both keratinocytes and fibroblasts and to promote their viability [26]. Similarly, glycerosomes were used to entrap *Thymus capitatus* [37] oil, targeting the treatment of oral cavity diseases and *Rosmarinus officinalis* [51] extract to enhance the stability of the antioxidant polyphenols.

Penetration enhancer-containing vesicles (PEVs) were developed using polyethylene glycol, for the enhancement of skin penetration of *Thymus* essential oil in oral cavity wounds [37]. The incorporation of activators such as ethanol and propylene glycol into the liposomes, as safe and nontoxic agents, is acceptable in pharmaceutical and drug formulations [72].

Glycethosomes are a subtype of liposomes (even a subtype of glycerosomes) developed by hydrating phospholipids with a mixture of water, glycerol, and ethanol. Glycerol is added due to its moisturizing and cosolvent properties and ethanol as a penetration enhancer, both helping the active substance pass through the skin. In a recent study, mangiferin glycethosomes have been developed for the adjuvant treatment of psoriasis. The results underlined their superior ability to promote the healing of an experimental wound induced by 12-O-tetradecanoylphorbol-13-acetate, confirming their potential application for the treatment of psoriasis or other skin disorders [46].

Hyalurosomes have shown a higher mechanical resistance than liposomes due to the sodium hyaluronate network, which stiffens the phospholipid vesicles. This ensures an increased residence time of the formulation at the site of action, avoiding drug leakage [73]. As regards the application in wound healing, previous studies reported the successful entrapment of *Glycyrrhiza glabra* [73] extract and *Citrus limon* var. *pompia* [26] extract in hyalurosomes and the development of curcumin gel-core hyalurosomes [74].

Marinosomes, or marine lipid-based liposomes, contain a high ratio of polyunsaturated fatty acids, omega-6, or omega-3. Among their main advantages, they influence membrane fluidity and have the ability to reduce oxidative stress, inflammation, and abnormal cell proliferation [75].

Sphingosomes, also called sphingomyelin liposomes, represent another VN composed of sphingolipids [76]. The natural sources of sphingolipids, mainly sphingomyelin, are mammal milk, egg yolk, and the brain. Sphingomyelin molecules are the most abundant sphingolipids also found in biological membranes, where they have structural functions and preferentially interact with cholesterol to form ordered domains called lipid rafts [77]. Sphingomyelin, a primary component of biomembranes, hydrolyzes and generates ceramides, effective in skin moisturization. Thus, sphingomyelin-based liposomes proved to increase the ceramide II level in a three-dimensional cultured human skin model [78].

**Figure 4:**
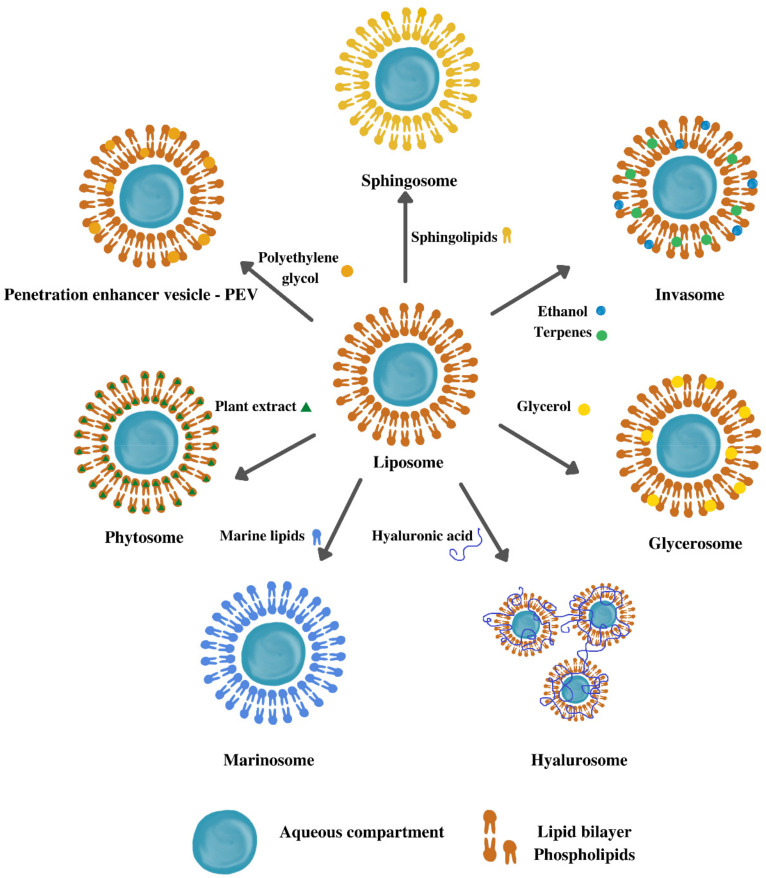
The main liposome derivates carriers.

The main characteristics of these liposome-derived vesicles are summarized in Figure 4.

To the best of our knowledge, several VNs, such as invasomes, marinosomes, and sphingosomes, bearing HEs or PCs have not been reported yet for wound-care applications. However, they have great potential to be studied in this regard.

### 5.2. Ethosomes

Ethosomes are vesicular multilamellar nanosystems, representing the third generation of elastic lipid carriers, that have a good ability to encapsulate drugs or HEs with different solubilities. These vesicles are composed of lipids, usually phosphatidylcholine, high concentrations of short-chain alcohols, in general between 20% and 50% ethanol, isopropyl alcohol, or propylene glycol up to 15%, cholesterol, and water. Ethanol, well-known as a permeation enhancer, confers softness, malleability, and ultra-deformability to the vesicle. Ethosomes have a smaller particle size as ethanol confers a negatively charged surface to the ethosomal vesicular system, good entrapment efficiency, and higher stability than traditional liposomes. Ethosomes are suitable for drug solubilization and improve skin delivery. The “ethanol effect” consists of its interspersion in the intercellular lipids, and changes within the dense alignment of the cell lipid layer, enhancing the lipid fluidity and decreasing the structural density of the lipid multilayer. Afterward, the “ethosomes effect” occurs, by the opening of new pathways due to the malleability and fusion of these nanovesicles with skin lipids, enhancing the inter-lipid penetration and permeation. Thus, the result consists of the deposition and release of PCs into the deep layers of intact or damaged skin, revealing the potential of ethosomes in wound-healing applications [11,12,42,61,79,80,81,82].

In wound-healing applications, ethosomes encapsulating *Fraxinus angustifolia* [27] extract, PCs such as curcumin [80], or drugs such as piroxicam [79] have been previously reported. Currently, ethosomes are primarily used for efficient topical delivery into deep layers of the skin or across the skin for both local and systemic delivery [11,12,42,61,79,80,81,82].

By the addition of surfactants in ethosomes, a novel type of carriers has been obtained, the transethosomes, which are considered the second generation of ethosomes or a subtype of transferosomes. They have been developed by combining ethanol and a surfactant, employed as an edge activator to improve the deformability of vesicles. The characteristics of transethosomes are the increased flexibility and the major transdermal potential [41,82,83] even though some authors reported negative effects on human skin, such as inflammation induced by these surfactant-based nanocarriers [59].

### 5.3. Niosomes

Niosomes are novel drug-delivery systems, also called non-ionic surfactant vesicles, that might entrap lipophilic drugs into vesicular bilayer membranes and hydrophilic drugs in the aqueous compartments. Therefore, the niosomes can encapsulate both lipophilic and hydrophilic PCs [21,39,84]. Niosomal carriers protect the therapeutic agent and improve its stability since the encapsulated drug is stored in a semi-biological environment with a longer shelf-life [39]. Niosomes are composed of non-ionic surfactants, such as Span™ 60, instead of phospholipids, being obtained by self-assembling non-ionic surfactants and cholesterol [50,85]. Thus, they allow the controlled delivery of active compounds and better penetration than other preparations commonly used nowadays, such as transdermal drug-delivery systems [21]. In comparison with liposomes, niosomes are known as cost-effective preparations with superior chemical and storage stability [50]. The experiments conducted by Priprem et al. evaluated a topical niosomal gel with anthocyanin complex extract, showing the enhancement of the topical delivery of the PCs. This formulation exhibited an anti-inflammatory effect and promoted oral wound closure in rats [85]. Niosomes encapsulating HEs (*Hypericum perforatum* [19], *Calendula officinalis* [47]) or propolis extract have been reported with promising results in wound-healing applications [21,39,50]. 

### 5.4. Transferosomes

Transferosomes are ultra-deformable phospholipid vesicles, being more elastic and deformable than conventional liposomes, due to the presence of an edge activator, usually a surfactant or another molecule capable of modifying the assembly of the bilayer. Thus, they can be prepared by adding Tween 80 to phospholipid vesicles, leading to the increase of the bilayer fluidity and the ability of the vesicle to squeeze through the inter-corneocyte matrix, and finally increasing the active substance deposition into deeper skin layers. In a recent study, *Myrciaria jaboticaba* [30] extract was successfully entrapped into transferosomes designed for the treatment of skin wounds and oxidative stress-related skin disorders [30].

### 5.5. Cubosomes

Cubosomes, or cubic-phase nanoparticles, have great potential as an alternative to the conventional lipid vesicles, liposomes. These hydrophilic surfactant systems could self-assemble as a bi-continuous cubic liquid crystalline phase [86,87]. Liquid crystalline nanoparticles are dispersions of liquid crystalline phases into a solvent, usually water, used in excess. Thus, cubosomes are liquid crystalline nanoparticles with the same unique properties of the bulk cubic phase but with a lower viscosity of the cubosome dispersion. The most investigated cubosomes are composed of binary systems of water and glyceryl monooleate. Since glyceryl monooleate is prone to lipolysis, the cubic phase is biodegradable [87,88,89].

**Figure 5:**
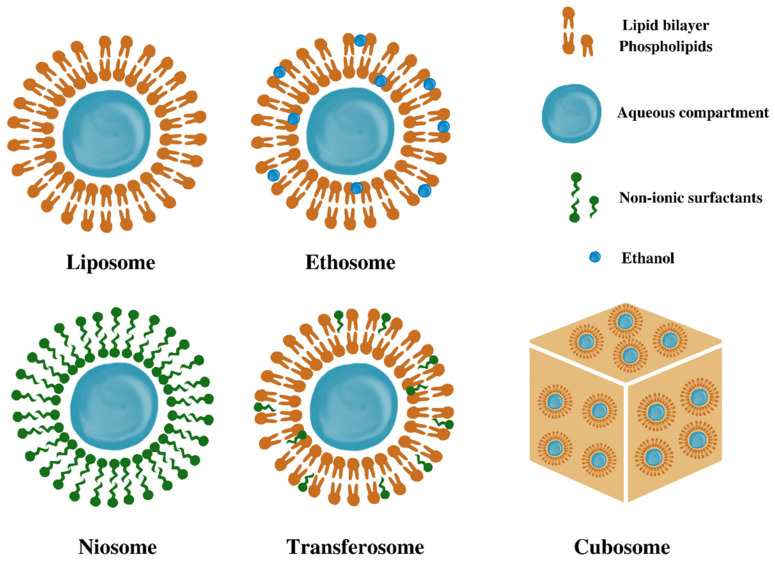
The main types of vesicular nanosystems with potential applications in wound healing.

Cubosomal systems are biocompatible, bio-adhesive, and biodegradable three-dimensional nanostructures with hydrophilic and hydrophobic domains, distinguished by their viscous nature, large surface area, and high ability to incorporate hydrophilic, lipophilic, and amphiphilic drugs. Concerning the applications of these vesicular systems, Thakkar et al. developed a silver sulfadiazine-entrapped cubosomal hydrogel with *Aloe vera* for topical treatment of infected burns, showing a superior healing rate compared to the cubogel with silver sulfadiazine alone [88]. Encouraging results for burn wound care were obtained by Morsi et al., who described the entrapment of silver sulfadiazine in cubosomes. The experimental data have shown better patient compliance and excellent healing results, with fewer side effects, than a commercially available product [87]. The main types of vesicular nanosystems with potential applications in wound healing are presented in Figure 5.

Table 1, Table 2, Table 3 and Table 4 summarize the studies investigating the development of VNs bearing HEs and PCs for wound healing.

## 6. Preparation Methods of Vesicular Nanosystems

All the methods of preparation of phospholipid vesicles are based on the phospholipidic property to form aggregated complexes when they are placed in an aqueous environment, because of their amphiphilic structures. In this way, hydrophobic parts avoid contact with the water molecules, while hydrophilic head groups remain in contact with the aqueous phase and are assembled in vesicular form. The underlying mechanism for the formation of the lipid vesicles is the hydrophilic–hydrophobic interactions and van der Waals forces between phospholipids and water molecules [106]. During the preparation of the VNs, the HEs and the PCs can be included in different steps of preparation, according to their solubility.

### 6.1. Hydration of Dry Thin Lipid Film: Bangham Method, Film Dispersion, or Thin-Layer Evaporation Method

In the first step, the phospholipids are dissolved in an organic solvent (chloroform, ethanol, methanol, etc.) in a round-bottom flask. In the second step, the organic solvent is completely evaporated under vacuum in a rotary evaporator, obtaining a thin film over the wall of the round-bottom flask. Afterward, the dried lipid film is hydrated with distilled water or isotonic PBS to form large multilamellar vesicles (MLVs). This process can be accelerated by using an ultrasound bath. The resulting large MLVs are subjected to sonication, finally obtaining small unilamellar vesicles (SUVs) [94,97].

The lipophilic PCs intended to be entrapped in vesicles can be dissolved in organic solvents together with phospholipids: *Myrciaria jaboticaba* [30] peel extract, *Narcissus tazetta* [84] extract, *Salvia triloba*, and *Rosmarinus officinalis* [102] essential oils, *Calendula officinalis* [70] extract, cinnamon oil [52], *Citrus limon* var. *pompia* [36] essential oil, or citral, ammonium glycyrrhizate [105], quercetin [95], curcumin [59], oryzanol [99], or asiaticoside [101], or with the surfactant for development of niosomes with *Calendula officinalis* [50] extract. Curcumin can also be dissolved in acetone and then mixed with phospholipid previously dissolved in the organic solvent [74]. Similarly, the shikonin solution was mixed with dendrimers solution, and then the phospholipids in organic solvents were added to obtain chimeric advanced drug-delivery nanosystems for shikonin, combining dendritic and liposomal technology [97].

Depending on their solubility, the PCs can also be added to the solutions used for film hydration. Thus, the thin film can be hydrated with the PBS HEs or PCs solution, such as *Carpobrotus edulis* [90] powder extract, anthocyanin complex [85], madecassoside [44], essential oil derivative alpha-bisabolol [99], with the ethanolic solution of the PC [74], with the aqueous solution of HEs (*Narcissus tazetta* extract [84]), or with the HE itself (Danggui Buxue extract [92], *Salvia aramiensis* extract [58], or *Aloe vera* leaf gel extract [91]). In some other cases, the extract can be added at the end and stirred with the vesicles dispersion (*Moringa oleifera* extract [29] and bromelain extract [94]).

### 6.2. Emulsion Evaporation

In this method, the lipids are dissolved in an organic solvent under mixing, and then slowly added to distilled water with gentle stirring and homogenized. The organic solvent is then removed by vacuum drying and the remaining solution is subjected to ultrasonication. Finally, the nanosystems’ dispersion is purified by centrifugation. This method was used to obtain astaxanthin-loaded nano-liposol [96].

### 6.3. Direct Sonication

VNs can be prepared by simple dispersion of the components (phospholipid and HE or PCs) in water or other solvents, or in the HEs itself for a few hours, then sonicated until a homogenous system with a small particle size is obtained [28]. Using this method, *Hypericum scruglii* extract [45], *Thymus capitatus* essential oil [37], *Fraxinus angustifolia* extract [27], *Azadirachta indica* [93] (neem) oil, curcumin [72], *Glycyrrhiza glabra* extract, or raw glycyrrhizin [73], resveratrol [104], oleuropein [7], phycocyanin [60], quercetin [25,98], and curcumin [25,100] were successfully incorporated.

The direct sonication of the dispersion containing phospholipids, HE, and water represents a green and fast preparation method since the use of organic solvents and dissipative processes are completely avoided [45,72]. Thus, a green method for the preparation of liposomes, without using an organic solvent, consisted of mixing the phospholipids and the lentisk oil with water, followed by a sonication step and purification through dialysis [40].

The HEs can also be dispersed in water or mixtures consisting of water/glycerol or water/sodium hyaluronate or water/glycerol/sodium hyaluronate to produce liposomes, glycerosomes, hyalurosomes, and gly-hyalurosomes, respectively, employed for hydration of the phospholipids, overnight, at room temperature; then, they are sonicated and purified by dialysis. In this way, *Citrus limon* var. *pompia* extract-loaded phospholipid vesicles were obtained [26]. Similarly, the phospholipids can be hydrated with the aqueous dispersion of HE [73].

In another study, a cold method was used to obtain *Achillea millefolium* extract-loaded ethosomes. Phospholipids were dissolved in ethanol and propylene glycol and HE was introduced with a syringe, followed by sonication [103].

### 6.4. Reverse Phase Evaporation Technique

In this technique, the lipids are dissolved in an organic solvent under mixing, and separately, the HE is dispersed in distilled water. Then, the HE is added to the solvent and homogenized. Finally, the suspension is heated in the water bath to evaporate the organic solvents and separated by centrifugation. In this way, *Hypericum perforatum* [19] extract-loaded niosomes were obtained [19].

### 6.5. Antisolvent Precipitation Technique

In this method of preparation, the lipids are dissolved in an organic solvent such as chloroform, and the aqueous solution containing the HE is slowly added to the lipophilic solution under vigorous stirring. The precipitate is separated by filtration or centrifugation and dried into a desiccator until complete solvent evaporation. By using this technique, *Aloe vera* whole extract and L-carnosine phytosomes were obtained [19].

### 6.6. Single-Step Injection Technique

Another preparation method consists of dissolving the active substance and the phospholipid in ethanol and propylene glycol under stirring. After this, the distilled water is directly injected into the previously obtained solution by using a microinjection pump or syringe. If a HE is used, after dissolving the phospholipids in ethanol and propylene glycol, the extract is injected slowly as a fine stream with a syringe in the solution and then the final volume is adjusted with distilled water. The resulting mixture is stirred and then sonicated to decrease the particle size [81,103].

After the preparation of the vesicle dispersion, it is subjected to purification by dialysis [7,25,100] or centrifugation [101]. The decrease in the particle size can be achieved by sonication, in most cases, or by extrusion in other cases [28,65,70,101,105]. In a study conducted by Paolino et al., another particular method was used, by applying alternative cycles of freezing and thawing and then extrusion to obtain unilamellar vesicles [101].

## 7. Characterization Methods of Vesicular Nanosystems

To ensure their in vitro and in vivo performance, VNs must be comprehensively characterized after their preparation, by evaluating their physicochemical properties [107]. Their attributes can affect the bulk properties, product performance, processability, stability, and appearance of the final product [106]. For an efficient active delivery at the wound site, the desirable properties of VNs are: small particle size, homogeneous (monodisperse) systems, good stability of the dispersion, high encapsulation efficacy, and optimal release profile of the actives [106,107].

### 7.1. Particle Size

Particle size and size distribution of lipidic nanocarriers are critical quality attributes for VNs, as well as essential components of stability. The particle size, expressed as average size or average diameter, is usually measured by laser Doppler electrophoresis [53] or dynamic light scattering [19,51,105]. The size of the vesicle mainly depends on the different concentrations of lipid and ethanol included in the formulation [103]. Extrusion, sonication, homogenization, and/or freeze–thawing techniques are used to control the size and the size distribution of different nanocarriers [106].

The smaller the particle size, the larger the surface-to-volume ratio is. The small size of vesicles is important for enhancing the cellular uptake or internalization, with particle size and polydispersity index (PDI) being the main physiochemical attributes that influence the endocytosis-dependent cellular uptake [106]. It is generally accepted that the particle size should be smaller than 300 nm to ensure efficient skin penetration and delivery of the payload into the deeper layers of the skin [103,106]. Nanovesicles with a diameter of 70 nm or below have shown maximum deposition of actives in both viable dermal and epidermal layers. Generally, lipid vesicles have diameters ranging from 40 to 800 nm, allowing them to attach to the lipid matrix of the stratum corneum and enhance the amount of active substances that penetrate the deeper layers of the skin [16].

### 7.2. Polydispersity Index

To measure the broadness of the size distribution and the degree of heterogeneity of particles, a dimensionless parameter is used: the polydispersity index, which has values between 0 and 1 [11,30]. According to the value of this index, the systems can be classified as monodisperse or polydisperse. The average diameter and polydispersity index of the vesicles can be determined by dynamic light scattering [37] or by photon correlation spectroscopy [26,36]. An acceptable dispersion is obtained when PDI has values lower than 0.3, indicating a homogenous liposomal population [39,103,106]. The PDI can be reduced by extrusion or sonication processes to obtain more uniform vesicles [11].

The physicochemical properties of lipidic nanocarriers, particularly PDI, influence their ability to accumulate in the target tissue. As a result, the creation of homogeneous (monodisperse) populations of nanocarriers of a specific size is required for the formulation of safe, stable, and efficient nanocarriers. Thus, controlling the particle size distribution should take into account the composition of the nanocarriers as well as the type of solvents and co-solvents used in their preparation [106].

### 7.3. Particle Shape

The morphology of the VNs can be analyzed by scanning electron microscope (SEM), transmission electron microscopy (TEM), and cryogenic TEM [41,45]. These microscopic methods can confirm the formation of lamellar vesicles, and suggest the impact of the addition of different ingredients to the composition of vesicles, showing the morphology, shape, and dimensions of the vesicles [41,53]. TEM involves the removal of the native environment of vesicles for preparation and may induce alterations in liposomal shape (shrinkage, swelling, and artifacts’ formation in the created image). The use of Cryo-TEM can overcome these limitations, keeping the vesicles close to their native state, and minimizing the shape distortion on shrinkage [107]. Moreover, information about the particle size and bilayer thickness can be obtained using Cryo-TEM images with the aid of the Fiji tool [28].

### 7.4. Zeta ζ-Potential (Surface Charge)

Zeta ζ-potential measurements are obtained through laser Doppler electrophoresis (measuring the electrophoretic mobility of particles) or dynamic light scattering [105]. The samples are suitably diluted beforehand to be optically clear and to avoid the reduction of scattered light that can be detected by the Zetasizer [30]. The zeta potential is caused by the net charge of the lipid structure in the formulation [6]. This is the main factor that indicates the general charge of a particle, and the repulsion or attraction forces between the particles. Thus, it provides a secondary parameter by which the stability of liposome dispersions is evaluated, by controlling the electrostatic interactions between the particles in suspension [21,51,107]. The negative charge of ethanol in the system prevents the aggregation of vesicles by electrostatic repulsion and resistance force. Moreover, negative zeta potential is responsible for higher percutaneous permeation of the drugs [11]. Differences in particle size provide information about aggregation and fusion phenomena. If the zeta potential of particles rises above −25 and +25 mV, it indicates an increase in stability, because they present repulsive forces that prevent the natural tendency to aggregate. The closer the potential value approaches to zero (uncharged particles), the faster the vesicles decompose by flocculation [21,107].

### 7.5. The Lipid Content of the Vesicle Dispersions

The lipid content of the vesicular dispersions could be assessed by the Stewart assay. An aliquot of vesicle suspension was added to a biphasic mixture of aqueous ammonium ferric thiocyanate solution (0.1 N) and chloroform. The concentration of the phospholipids can be obtained by measuring the absorbance of the organic phase. The aggregation efficiency (AE%) can be calculated as the effective amount of aggregated phospholipids, expressed as the percentage of the amount initially used [98].

### 7.6. Phase Behavior

Differential scanning calorimetry (DSC) may be used to confirm the formation of the vesicles, but also to investigate the presence of potential interaction or conformation between components of the vesicular systems. On the DSC thermograph, endothermic or exothermic peaks appear, which show the ingredients’ degradation, and the differences between the thermographs of individual components and the mixture of them provides information about interactions or complex formation [19,71,95].

DSC also determines the transition temperature of the lipid bilayer, an important parameter that influences the fluidity of the liposomal lipid bilayer and may affect liposomal properties such as aggregation, fusion, and stability. Transition temperature may also be investigated by Fourier transform-infrared spectroscopy (FTIR) or X-ray diffraction studies (XRD) [107].

### 7.7. Entrapment Efficiency

Loading the right amount of active substance in VNs is crucial to achieving therapeutic efficacy [107]. The entrapment efficiency or encapsulation efficiency (EE%) is represented by the delivery potential of the VN that is directly associated with its drug-carrying capacity [11]. Briefly, EE% represents the amount of drug entrapped in the VN, and may be calculated by using the following equation [108]:

EE% = Entrapped drug concentration/Total drug concentration × 100 (1)

The vesicular dispersion is placed into a dialysis cassette and further dialyzed, centrifuged, and then the supernatant is analyzed [44,53]. The quantification of the PCs is performed by specific methods, such as gas chromatography in the case of volatile oils, high-performance liquid chromatography with diode array detection (HPLC-DAD), or UV-Vis spectroscopy for other actives [39,40,53,92].

The EE% of the PC in the vesicles can be correlated with the antioxidant activity, reported as a percentage ratio between the antioxidant activity of the samples before and after the purification process (dialysis), measured by the DPPH colorimetric test and calculated according to the following equation [7,30]:

Antioxidant activity% = [ABS_DPPH_ − ABS_sample_)/ABS_DPPH_] × 100 (2)

The vesicle composition, the method of preparation, as well as the rigidity of the bilayer membrane, can have a crucial impact on the EE of a certain drug [107]. In the case of ethosomes, it is mentioned that the quantity of ethanol and phospholipids influences the encapsulation of PCs in a positive manner. Taking this into consideration, the ethanol concentration is usually up to 40%, as its fluidization effect at higher concentrations may cause leaking of HE from the lipid bilayer, subsequently with a decrease of EE% [11].

### 7.8. In Vitro Drug Release of the Active Substance

An appropriate release of PCs entrapped in VNs is mandatory for the efficacy of the product in wound healing. The aim is to achieve a sustained release of the active substances at the site of the injury and prolongation of their effect to speed up the wound-healing process [16].

In the development of liposomes for the controlled release of actives, the results obtained from the in vitro release studies are extensively accepted as an extrapolation to in vivo performance of liposomes [107].

The release of PCs from VNs can be analyzed by the dialysis method or Franz diffusion cells by using various release media. Samples are withdrawn from the release compartment at specific time intervals, with the volume being replaced with fresh release medium to maintain sink conditions. The amount of PC released can usually be determined by using UV-Vis spectrophotometry [72,97], HPLC methods [58,95,102], or measuring the antioxidant activity with the DPPH colorimetric test [7,45] or total phenolic content [73]. The percentage of released phytocompounds is calculated from the cumulative drug amount released at different time intervals, using the following equation [74,102]:

Release% = [(total amount of drug − detected amount of drug) × 100/total amount of drug] (3)

The release of phytocompounds from the topical formulation containing the vesicle dispersion can also be determined. Moreover, supplementary information about the kinetics of active release can be obtained [21].

### 7.9. Physical Stability

The liposomal colloidal dispersion systems are thermodynamically unstable, being prone to aggregation, flocculation, and fusion, or the entrapped substance may precipitate during storage [44]. The verification of particular characteristics, such as chemical and physical stability, the size and structural conservation, the maintenance of encapsulated drugs, and the impact of biological fluids on liposomal properties, are all part of the evaluation of liposomal stability [107].

Mainly, the physical stability of liposomes is influenced by the temperature. This can be studied by monitoring the aggregation or fusion of liposomes and the leakage of the active substance. The turbidity assay is a practical approach to determine the relative size of liposomes and reflects the stability of the liposomes. This parameter is determined spectrophotometrically, with the increase of the absorbance values being correlated with the particle size increase [44]. The physical stability of the vesicles in the dispersion is evaluated by monitoring the average size, the polydispersity index, and the zeta potential for 1 month [53,102], 2 months [37,45], 3 months [30,40], 4 months [7], or 6 months [41] of storage at room temperature.

### 7.10. Leakage Rate

The leakage rate evaluates the performance of liposomes during their storage and represents the ratio of encapsulation efficiency during the storage period (Wi) to initial encapsulation efficiency (W0). The leakage rate can be measured four weeks after preparation [44,92]. This parameter can be calculated using the following equation [44]:

Lr% = (1 − W_i_/W_0_) × 100 (4)

### 7.11. Chemical Stability

Chemical stability refers to liposomes’ capacity to maintain EE% levels when changes in the medium occur, such as pH alterations, electrolyte composition, oxidizing agents, and the presence of surface-active chemicals. As the lipids are prone to suffer oxidative reactions, their chemical degradation may induce permeability changes within the lipid membrane. Furthermore, the interactions between the entrapped drugs and phospholipids can also interfere with vesicles’ chemical stability [107].

### 7.12. Other Characterization Methods

Fourier transform-infrared spectroscopy is performed for the analysis of functional groups of HE-loaded vesicles [103]. The analysis is performed for each main component and their physical mixture by using an FT-IR spectrometer [19].

X-ray diffraction studies are performed to record X-ray diffractograms for each main component and their physical mixture [19,98]. Small or wide-angle X-ray scattering analysis allows for obtaining relevant structural parameters on bilayer-based structures, e.g., vesicles and lamellar phases [25,100]. The vesicular dispersions are loaded into glass capillaries and the diffraction patterns are recorded, usually at 25 °C, followed by a calculation of the electron distance distribution and the bilayer electronic density profile. Through this method, relevant structural parameters are obtained, as well as the distribution of electron density in the polar and a-polar regions of membranes [98].

## 8. Methods of Evaluation of Vesicular Nanosystems for Wound Healing

### 8.1. In Vitro Skin Delivery of the Active Compounds

The analysis of the permeation of PCs is performed by using Franz diffusion cells. The receptor compartment is usually filled with saline or PBS and the samples of PC- or HE-loaded nanosystem suspension are applied onto the membrane surface. An exact amount of the receiving medium is withdrawn at different time intervals and the content in PCs is assessed by specific quantification methods (e.g., HPLC or UV-Vis spectroscopy). The extracted amount is replaced with fresh medium to maintain a constant volume in the receptor compartment [25,100,103,105]. 

The skin retention of PCs may also be analyzed using Franz diffusion cells, in ex vivo studies. Skin samples are collected and placed between receptor and donor compartments of Franz diffusion cells. The tested product is placed onto the skin, in the donor compartment. The quantity of PCs accumulating in different skin layers, expressed as an amount in μg/cm^2^ and as a percentage of the total PC amount applied on the skin, may be determined in the stratum corneum by tape stripping and in the epidermis and dermis after their separation [99].

### 8.2. Cellular Uptake by Vesicles

After skin application of the vesicular systems, particularly when the skin is damaged, an increased local bioavailability and PCs’ residence time in the injured tissue is obtained due to its internalization in the cells. Therefore, an increase in the activity of the PC is observed. A membrane marker is used to label the vesicles and a fluorescent molecule to identify the aqueous cell compartment. The cellular uptake of the active compounds is then studied by confocal microscopy after the incubation of different skin cells with PC-loaded vesicles. The distribution of the fluorescent-labeled vesicles into the cells is checked [27,61,99].

### 8.3. Cell Viability Studies

The viability of cells is analyzed to indicate the biocompatibility of the formulation [30]. Cytotoxicity can be analyzed using MTT (3-[4, 5-dimethylthiazol-2-yl]-2, 5 diphenyl tetrazolium bromide) by a colorimetric assay. Results are shown as percent of cell viability in comparison with non-treated control cells (100% viability) [27,29].

### 8.4. Scratch Assay—In Vitro Wound Healing Effect, Cell Migration Assay

The in vitro scratch assay is performed using a monolayer of cells to analyze the ability of the HE in aqueous dispersion or incorporated into vesicles to stimulate the cell’s proliferation and migration. The cells are seeded in plates and a mechanical scratch wound is produced in the confluent cell monolayer. The percentage of wound closure (wound healing %) is calculated by measuring the lesion areas or according to cell migration in the scratch area by using a computer program [30,70]. The percentage of migration can be calculated by using the following equation:

Percentage of migration = (A_0_ − A_n_)/A_0_ × 100% (5)

where A_0_ is the initial area of the scratch site, and A_n_ is the area of the scratch site after the nth hour [29].

### 8.5. In Vivo Wound Healing Effect on Animal Model

The wound healing process after the topical application of the dosage forms may be measured at different time intervals. Usually, the in vivo efficacy test is evaluated on an experimental animal model after performing surgical procedures to create identical skin defects. The wounds are photographed at preset intervals of time (days), and then the wound area is measured using specific software. The percentage of wound reduction area is calculated with the following equation [80]:

Wound reduction area% = [(Wound area day 0 − Wound area relative day)/Wound area day 0] × 100 (6)

The histopathological examination of the skin tissue samples may be assessed using a scoring system. For example, Abramov’s method uses a score of 0–3 for each section for inflammatory cells, collagen deposition, angiogenesis, granulation tissue formation, and epithelialization [80].

Methods of characterization and other analyses undertaken on VNs bearing HEs or PCs are presented in Table 5, together with the results of each study.

## 9. Topical Delivery Systems Containing Herbal Extracts

Current treatment options for wounds include more than 3000 wound dressings. Various options from traditional dressings such as gauze to film, hydrogel, hydrocolloid, foam, hydrofiber, alginate, biological, and composite dressings, are available. Lately, modern approaches such as 3D-printed dosage forms and drug-delivery devices were developed. The main challenges of conventional wound care are microbial infection, inflammation, and inadequate blood supply. In the past decades, advanced approaches such as oxygen therapy, negative-pressure wound therapy, or gene therapy were developed, but their clinical applications are limited by the high cost or the need for special technologies.

Given the high diversity of wound care products, several classifications based on different criteria, such as clinical performance, physical form, or the source of the material, have been proposed. According to the FDA classification, wound-care products are classified as drugs, medical devices, biological products, or combination products. Conventional wound healing pharmaceutical formulations include liquid (solutions, suspensions, or emulsions) and semisolid (creams or ointments) products. Wound care devices may be Class I, II, III, or unclassified [109,110,111].

Lipid nanovesicles can be included in different topical systems, such as powders, gels, solutions, creams, ointments, and dressings, to facilitate the application to the wound. Targeted action of the formulation along with the sustained release of the active substances are considered the principal requirements in chronic wound healing. These criteria can be successfully fulfilled by using the lipid vesicles included in the appropriate formulations [112].

Many studies describe the assessment of the vesicular system after its incorporation into a gel vehicle, for a suitable topical application of formulation [21,80,85,92,103]. Hydrogels or hydrated polymer dressings are the most often employed gels for cutaneous application. They contain a high amount of water in a gel base, help to regulate the fluid exchange from the wound site, and confer to them a soft consistency, similar to the natural tissue. They also provide moisture to the wound, important to promote granulation, epithelialization, and autolytic debridement. The high water content of hydrogel dressings can cool the wound, producing pain relief that can last up to 6 h, and contribute to their biocompatibility and rehydrating capacity. Dressing-change discomfort is also reduced because hydrogels do not adhere to the wound surface due to low interfacial tension between the hydrogel surface and the body fluid. Additionally, the elastic nature of hydrated hydrogels minimizes irritation to the surrounding tissues. Hydrogels facilitate autolytic debridement, fill in the dead space from the surface of the wound, and can be used even when the infection is present [87,88].

An actual interest is the development of modern dressings (films, hydrogels, hydrocolloids, wafers, membranes, sponges, foams, etc.). These dressings cover the wound and ensure a moist environment, which is necessary for rapid and efficient healing, with reduced scar complications. Moreover, these dressings ensure non-adhesion to the injured area and do not cause trauma when removed. For chronic wounds and those with impaired healing, such as bedsores, venous ulcers, or diabetic foot ulcers, it has been found that wound dressings’ application has significantly reduced the cost of the treatment. Modern dressings can incorporate and deliver active substances, such as antimicrobial, anti-inflammatory, or analgesic agents, at the wound site [94,113,114]. Including VNs in modern dressings can be an interesting field of research, because this may provide targeted and sustained delivery of constant concentrations of the drug on the wound site together with the improved penetration of the drug, swelling in the presence of wound exudates, better protection of active ingredients, and thereby favor the wound healing [112]. Recent studies highlighted that modern dressings with natural active compounds or HEs can be an ideal delivery system in wound healing [115,116]. In addition, other studies describing the incorporation of lipid vesicles in modern dressings for wound healing have shown promising results [117,118,119,120]. Although, a thorough search of the relevant literature yielded only a limited number of studies in which VNs with PCs were included in polymeric dressings for wound healing [31,94]. Table 6 presents VNs bearing HEs or PCs incorporated in topical systems.

## 10. Conclusions

As a response to the increasing prevalence of acute and chronic wounds, currently, a high number of studies focus on formulating innovative and effective healing products. This paper highlighted the preparation and characterization of lipid vesicle nanocarriers bearing HEs or PCs for wound healing, by emphasizing the main types of phospholipid nanovesicles, together with the challenges of the entrapment of the PCs or HEs in those nanosystems. The main outcomes of recent studies investigating VNs loaded with HEs or PCs are presented, as a basis that might help researchers in future studies. In the future, the development of novel wound dressings containing vesicular nanocarriers with PCs is expected to gain importance due to their multiple advantages over traditional topical products.

## Figures and Tables

**Table 1:** Vesicular nanosystems as carriers of herbal extracts with wound-healing effects.

Entrapped HEs	Method of Extraction	Active Compounds	Components of VNs	VNs’ Preparation	Ref.
**Liposomes**					
*Carpobrotus edulis* powder extract	Fresh leaves homogenized in distilled water, frozen, and lyophilized	Flavonoids	Hydrogenated phosphatidylcholine, cholesterol	Dry thin-film method	[90]
*Pistacia lentiscus* oil	Marketed product	Fatty acids, phenolic compounds ^a^	Soy lecithin	Hydration, sonication	[40]
*Aloe vera* leaf gel extract	Fresh gel homogenized, frozen, defrosted, centrifugated	Glycoproteins, aloesin	Soy lecithin	Bangham method, mechanochemical method	[91]
*Angelica sinensis* and *Astragali radix* ratio 1:5	Decoction	Ferulic acid coupled with astragaloside IV	Soy phosphatidylcholine, cholesterol	Thin-film dispersion, ultrasonic method	[92]
*Salvia aramiensis* aerial part extract	Methanol, ethanol extraction in shaking bath, water extraction-infusion	n.a.	Dipalmitoylphosphatidylcholine, cholesterol	Dry thin-film hydration	[58]
**Liposomes and hyalurosomes**					
*Azadirachta indica* oil (Neem oil)	Marketed product	Glycerides, fatty acids, sulfur-containing compounds, flavonoids ^b^	Soy lecithin, argan oil, sodium hyaluronate	Direct sonication	[93]
*Glycyrrhiza glabra* root extract	Percolation in ethanol	Glycyrrhizin-triterpenoid saponin glycoside, polyphenols	Soy phosphatidylcholine, Phospholipon 90G, Sodium hyaluronate LMW	Hydration, sonication	[73]
**Niosomes**					
*Calendula officinalis* flowers and leaves extract	Methanol extraction	Steroids, terpenoids, triterpenoids, phenolic acids, flavonoids, carotenes	Tween 60, cholesterol	Dry film hydration, sonication	[50]
*Hypericum perforatum* flowering tops	Ethanol extract, DIG–MAZ multifunctional extraction system	Hyperforin, hypericins, flavonoids	Span 20, 60, 80, cholesterol	Modified reverse phase evaporation	[21]
**Transferosomes**					
*Myrciaria jaboticaba* fruits peel	Pressurized hot water extraction	Flavonoids, anthocyanins, ellagitannins	Lipoid S75, Tween 80, hydroxyethylcellulose, Sodium hyaluronate	Hydration, sonication	[30]
**Phytosomes**					
*Moringa oleifera* leaves extract	Maceration, ultrasound-assisted extraction	Quercetin, kaempferol, chlorogenic acid, rosmarinic acid	l-α-lecithin, cholesterol, polysorbate 80	Thin-film hydration, sonication	[29]
**AuNP–phytosomes and liposomes**					
*Calendula officinalis* flowers extract	Methanol for 6 h, at room temperature	Chlorogenic acid, quercetin	Egg phosphatidylcholine, cholesterol	Thin-film hydration, extrusion	[70]
**Ethosomes and PEVs**					
*Fraxinus angustifolia* leaves and bark extract	Extraction in ethanol (1:4 *w/v*) under continuous stirring at room temperature, for 24 h.	Polyphenolic compounds ^c^	Phospholipon50, Transcutol P, ethylene glycol triglycerides, fatty acids	Hydration, sonication	[27]
**Glycerosomes, liposomes, gluglycerosomes, gel-gluglycerosomes, hyal-glulycerosomes**					
*Hypericum scruglii* aerial parts extract	Marketed product	Naphthodianthrones ^d^, phloroglucinols ^e^, phenolic acids, flavonoids ^f^, xanthones, terpenes	Phospholipid S75, glycerol, dextrin, gelatin, hyaluronan	Hydration, sonication	[45]

Legend: ^a^ tocopherols, carotenoids, and anthocyanins; ^b^ quercetin, kaempferol, muricetine; ^c^ quercetin, catechin, rutin, tannic acid; ^d^ hypericin, pseudohypericin; ^e^ hyperforin, adhyperforin; ^f^ hyperoside, rutin, quercitrin. LMW—low molecular weight; n.a.—data not available; PEVs—penetration enhancer-containing vesicles; HE—herbal extract; PC—phytocompound.

**Table 2:** Vesicular nanosystems as carriers for phytocompounds with wound-healing effects.

Entrapped PC	Source of PCs	Components of VNs	VNs’ Preparation	Ref.
Liposomes				
Bromelain extract	*Ananas comosus*	Egg phosphatidylcholine, cholesterol	Thin-film hydration	[94]
Madecassoside	*Centella asiatica*	Egg yolk lecithin, cholesterol	Thin-film hydration	[44]
Curcumin	*Curcuma longa*	Lecithin, cholesterol, propylene glycol	Hydration, sonication	[72]
Quercetin	Various species	Phosphatidylcholine, cholesterol	Thin-film hydration, sonication	[95]
Curcumin	*Curcuma longa*	Phospholipon 90G, oligochitosan (used for coating liposomes)	Thin-film hydration, sonication	[59]
Usnic acid	Lichens-*Cladonia substellata*	Phosphatidylcholine (Lipoid GMBH 75%)	Thin-film hydration	[31]
**Nano-liposol**				
Astaxanthin	Yeast, algae, and otheraquatic species	L-α-phosphatidylcholine from soybean	Modified emulsion evaporation method	[96]
**Liposomal locked-in dendrimers**				
Shikonin	Species of genera *Alkanna*, *Lithospermum*, *Echium*, *Onosma*, *Anchusa*	Egg phosphatidylcholine	Thin-film hydration	[97]
**Santosomes**				
Phycocianin	Blue-green algae	*Santolina insularis* essential oil, hydrogenated phosphatidylcholine, propylene glycol	Hydration, sonication	[60]
**Liposomes and PEVs**				
Quercetin	Fruits, vegetables ^a^	Lipoid S75, PEG 400	Hydration, sonication	[98]
Quercetin and curcumin	Various species	Lipoid S75, octyl-decyl polyglucoside, PEG 400	Hydration, sonication	[25]
**PEVs**				
Oryzanol and alpha-bisabolol	Oryzanol-rice bran oil and alpha bisabolol-Chamomile essential oil	Phospholipid (Epikuron 200), penetration enhancers (labrasol, transcutol)	Thin-film hydration	[99]
**Phytosomes**				
Sinigrin	Brassicaceae family	l-α-phosphatidylcholine hydrogenated (soybean)	Thin-film hydration	[71]
**Ethosomes**				
Curcumin	*Curcuma longa*	Egg lechitin, cholesterol	Ethanol injection, sonication	[80]
**Hyalurosomes**				
Curcumin	*Curcuma longa*	Enriched soy phosphatidylcholine (Phospholipon 90G)	Hydration, sonication	[100]
**Gel-core hyaluosomes**				
Curcumin	*Curcuma longa*	Lipoid S100, Hyaluronic acid, Tween 80, Poloxamer 407	Thin film evaporation, extrusion	[74]
**ULs**				
Asiaticoside	*Centella asiatica*	Fully saturated pure lecithin, saturated/unsaturated lecithins (Phospholipon 100G), sodium cholate	Thin-film hydration	[101]
**Transferosomes, glycoltransferosomes**				
Mangiferin	Various plants: mango leaves, fruits, by-products (e.g., peel, kernel seed)	Soy lecithin, glycerol, propylene glycol, Tween 80, mucin	Mangiferin dispersed in hydrating blend ^b^ sonicated, added in phospholipid and Tween 80, sonicated	[41]
**Collagen-enriched transferosomes, glycerosomes, and glytransferosomes**				
Oleuropein	Olive oil	Lipoid S75, collagen, Tween 80	Direct sonication	[7]

Legend: ^a^ apples, berries, onions; ^b^ water, glycerol, propylene glycol. PEVs—penetration enhancer-containing vesicles; ULs—ultra-deformable liposomes; HE—herbal extract; PC—phytocompound.

**Table 3:** Vesicular nanosystems including herbal extracts with potential benefits in the treatment of wounds.

Entrapped HEs	Active Compounds	Effect of HEs	Method of Extraction	Components of VNs	VNs’ Preparation	Ref.
**Liposomes**						
*Salvia triloba* and *Rosmarinus officinalis* essential oils	Eucalyptol and camphor	Antioxidant, anti-inflammatory, antibacterial	Marketed product	Phospholipon 90G, cholesterol	Dry thin-film hydration	[102]
*Citrus limon* var. *pompia* essential oil or raw citral	Terpenes-citral	Antibacterial activity	Citral/ essential oil-steam distillation	Lipoid S75	Hydration, sonication	[36]
Cinnamon oil	Essential oil	Antimicrobial effect	Marketed product	Soy lecithin and cholesterol	Thin-film hydration	[52]
**Glycerosomes**						
*Rosmarinus officinalis* leaves extract	Polyphenolic compounds ^a^	Antioxidant, antimicrobial	24 h stirring at room temperature with 70% ethanol	Phosphatidylcholine, glycerol	Hydration of phospholipids -Mozafari method	[51]
**Liposomes, glycerosomes, PEVs**						
*Thymus capitatus* essential oil	Carvacrol	Antimicrobial effect	Extraction with circulatory Clevenger-type apparatus	Soy lecithin, water/glycerol, water/propylene glycol	Hydration, sonication, dialysis	[37]
**Glycerosomes, hyalurosomes, gly-hyalurosomes**						
*Citrus limon* var. *pompia* fruits	Flavones ^b^	Anti-inflammatory, antioxidant	Sonication, hydroethanolic extract	Lipoid S75, sodium hyaluronate	Hydration, sonication, dialysis	[26]
**Ethosomes**						
*Achillea millefolium* antenna parts	Flavonoids, caffeic acid derivatives	Antibacterial, antioxidant, wound healing	Maceration with 70% ethanol	Phospholipid, ethanol, propylene glycol	Cold method, sonication	[103]
**Phytosomes**						
*Aloe vera* dry extract ^c^	Acemannan, β-sitosterol, glycosides (aloins), anthraquinone (aloe emodin)	Anti-inflammatory, antioxidant, cytoprotective, ↑ VEGF expression, ↑ NO synthesis	Marketed product	Soy lecithin	Antisolvent precipitation technique	[19]

Legend: ^a^ rosmarinic acid, carnosic acid, carnosol; ^b^ naringin, neoeriocitrin, neohesperidin; ^c^ co-encapsulation of L-carnosine/*Aloe vera* extract. VEGF—vascular endothelial growth factor, NO—nitric oxide; HE—herbal extract; PC—phytocompound.

**Table 4:** Vesicular nanosystems including phytocompounds with potential benefits in the treatment of wounds.

Entrapped PC	Effect of PCs	Source of PCs	Components of VNs	VNs’ Preparation	Ref.
**Invasomes**					
Terpenoids ^a^	Antibacterial, anti-inflammatory	Oil fraction of various plants	Soybean lecithin	Mixing the terpenoid with ethanol and phospholipids, extrusion	[28]
**Liposomes and niosomes**					
Resveratrol	Antioxidant	Grapes, nuts, berries	Soy phosphatidylcholine (Phospholipon 90G), glycerol monooleate, polyglyceryl-3 dioleate	Direct sonication	[104]
**ULs**					
Ammonium glycyrrhizate	Anti-inflammatory	*Glycyrrhiza glabra*	Soy phosphatidylcholine (Phospholipon 90G)	Thin-film hydration	[105]

Legend: ^a^ thymol, menthol, camphor, and 1,8-cineol. ULs—ultra-deformable liposomes; HE—herbal extract; PC—phytocompound.

**Table 5:** In vitro and in vivo studies undertaken for vesicular nanosystems loaded with herbal extract or phytocompounds.

Entrapped HE or PC	In Vitro Release Study/In Vitro Skin Permeation Study	Cell Culture Studies	In Vivo Studies	Main Results	Ref.
**Liposomes**					
*Carpobrotus edulis* powder extract	No/No	No	Male Wistar-albino rats	Positive effects on the healing process in both incisional and excisional wound tissues	[90]
*Pistacia* *lentiscus* oil	No/Franz cells, pig skin	HaCaT, primary mouse embryonic fibroblasts (3T3)	No	Stability of the system in dispersion, ↑ the PCs in the skin, ↑ the ability to counteract damages induced by oxidative processes, beneficial effect on lesion regeneration and healing	[40]
*Aloe vera* leaf gel extract	No/No	NB1RGB cells, NHEK(F) cells	No	↑ the cell proliferation and collagen synthesis, ↑ bioavailability of the HE, ↑ skin properties	[91]
*Angelica sinensis* and *Astragali radix* ratio 1:5	No/No	No	Adult male Sprague-Dawley rats	↑ therapeutic efficacy, ↑ wound closure; histological improvements, ↑ hydroxyproline levels; ↑ CD34, KI67, COL1α1, COL3α1 expression levels in wound granulation tissues compared to control groups in vivo, ↑ VEGF/PI3K/AKT and TGF-β/SMADS signaling pathways, which might contribute to the ability to ↑ full-thickness excisional wound healing in rats	[92]
*Salvia aramiensis* aerial part extract	Franz cells, dialysis membrane/No	L929 cell line (mouse fibroblast)	No	Strong antioxidant effect and potential wound-healing activity	[58]
Bromelain extract	No/No	No	Male Wistar rats	Absence of edema on the 14th day in animals treated with bromelain entrapped in nanocarriers	[94]
Curcumin	Dialysis, dialysis membranes/No	HDF	Male Wistar rats, New Zealand rabbits	Monodispersity and no vesicle aggregation even in long-term storage, considerable wound-healing properties in the early stage, antibacterial activity on burn wounds similar to SSD cream application	[72]
Quercetin	Diffusion cells system /No	No	No	Acceptable stability, biphasic pattern of drug release behavior	[95]
Curcumin	No/No	3T3 cells (mouse fibroblasts)	Mice *Mus musculus* var. albino.	↑ healing rates, ↑ scar treatment effects by incorporation in liposomes, compared to native curcumin, ↑ wound healing, ↑ scar treatment effect of curcumin liposomes compared to curcumin nanoplexes	[59]
Usnic acid	No/No	No	Male *Rattus norvegicus albinus*, Wistar lineage	↑ burn healing, probably related to the modulation of the inflammatory response, epithelialization, and collagen formation	[31]
*Salvia triloba* and *Rosmarinus officinalis* E.O.s	Dialysis, permeable bag membrane/No	No	No	Preservation of antioxidant properties of E.O. constituents, ↓ anti-inflammatory activity of the pure E.O. ↑ activities for the liposome-encapsulating E.O.	[102]
*Citrus limon var. pompia* E.O. or raw citral	No/No	HaCaT	No	Citral-loaded liposomes more effective than pompia E.O. liposomes in counteracting the growth of bacteria (*E. coli* and *S. aureus*) and fungi (*C. albicans*)	[36]
Cinnamon oil	No/No	No	No	↑ E.O. stability by liposome encapsulation ↑ antibacterial activity on MRSA and MRSA biofilms, ↑ antibiofilm activities and active time of liposome-encapsulating E.O. compared to free E.O.	[52]
**Nano-liposol**					
Astaxanthin	No/No	NIH 3T3 fibroblast cells	No	%EE ↑ of astaxanthin, good stability ↓ ROS, ↑ wound healing of fibroblasts without cytotoxicity	[96]
**Liposomal locked-in dendrimers**					
Shikonin	Dialysis, dialysis sacks/No	No	No	Adequate drug encapsulation, advantageous release profiles, satisfactory stability of liposomal formulations	[97]
**Liposomes and hyalurosomes**					
*Glycyrrhiza glabra* root extract	Dialysis, tubing Spectra/Por^®^ membranes/No	Primary mouse embryonic fibroblasts (3T3)	Female CD-1 mice	↑ effect of licorice extract, especially when delivered from hyalurosomes, ability to retain the extract components over time, ↑ in vitro and in vivo biological activity	[73]
**Liposomes and hyalurosomes**					
Neem oil (*Azadirachta indica* oil)	No/No	HaCaT and fibroblasts (3T3)	No	↑ biocompatibility, effective protection of the skin cells from oxidative stress, ↑ efficacy of the oil; argan-hyalurosomes → more viscous, more suitable for skin application	[93]
**Liposomes and PEVs**					
Quercetin and curcumin	No/ Franz cells, pigskin	No	Female Hsd:ICR(CD-1) mice	↑ Anti-inflammatory activity → inhibition the onset of skin wounds during TPA treatment; protective effect, more relevant in curcumin PEG-PEV formulation, ↑ drug bioavailability in the target tissue	[25]
Quercetin	No/No	3T3 mouse fibroblasts, cellular uptake	Female cd-1 mice	In vitro studies—massive uptake and diffusion of PEVs in dermal fibroblasts; in vivo studies—amelioration of the tissue damage in TPA-inflamed skin, attenuation of edema and leukocyte infiltration, especially using 5% PEG-PEVs	[98]
**PEVs**					
Oryzanol and alpha-bisabolol	No/Franz cells, dorsal rat skin- ex vivo deposition/permeation	No	Male Wistar rats	Favorable properties in terms of size, charge, stability, skin deposition for studied PEVs; faster onset, superior wound healing for oryzanol and alpha-bisabolol-loaded PEVs compared to a marketed product; early signs of neo-angiogenesis and collagen production compared to groups treated with PEVs loaded with oryzanol only or the marketed product	[99]
**ULs**					
Asiaticoside	No/Franz cells, adult human skin	Primary human dermal fibroblasts	Rats—Male Sprague-Dawley	↑ asiaticoside permeation through human SCE, ↑ intracellular drug delivery into primary human fibroblasts, significant ↑ collagen biosynthesis both in vitro and in vivo compared to the simple aqueous drug solution.	[101]
Ammonium glycyrrhizate	Franz cells, human SCE/synthetic membrane	No	Human volunteers	Biocompatible, deformable, allowed passage of ULs, delivery of A.G. in specific skin layers, pseudo-zero-order release kinetic, 50% of the entrapped drug is released in 24 h—potential depot effect of ULs in the skin; ↑ anti-inflammatory activity of drug of 15- and 30-fold compared to equivalent topical application of A.G. solution on healthy volunteers, time-dependent effect	[105]
**Transferosomes**					
*Myrciariajaboticaba* fruits peel	No/No	HaCaT	No	↓ H_2_O_2_ damage induced in cells, ↑ wound healing in HaCaT	[30]
**Transferosomes, glycoltransferosomes**					
Mangiferin	No/No	Mouse embryonic fibroblasts (3T3)	Female CD-1 mice	Optimal performances of mangiferin delivery, ↑ wound-healing properties; cytocompatibility, protection of fibroblasts from oxidative stress, ↑ proliferation, migration, wound closure in vitro; protection of mouse skin from chemically induced injury in vivo, ↓ inflammatory infiltration; glycoltransferosomes and mucin-glycoltransferosomes, ↑ deposition of mangiferin in epidermis and dermis; ↑ ability to pass across the biological membranes	[41]
**Collagen-enriched transferosomes, glycerosomes, and glytransferosomes**					
Oleuropein	Dissolution tester/No	Mouse embryonic fibroblasts, keratinocytes	No	↑ woundhealing efficacy, ↓ production of NO along with the damage induced by ROS, especially when cells were treated with collagen-enriched vesicles	[7]
**Hyalurosomes**					
Curcumin	No/ Franz cells, pigskin	HaCaT	No	↑ physicochemical properties, ↑ biological performances by using sodium hyaluronate dispersion as a hydrating medium of phospholipids; immobilization of vesicles by hyaluronan → ↑ EE%, stability, rheological properties, local drug availability, therapeutic activity, in vivo fast healing process	[100]
**Gel-core hyaluosome**					
Curcumin	Dialysis/No	No	Female Sprague-Dawley rats	↑ curcumin skin penetration, dermal localization, protection against degradation, ↑ healing, ↑ histological progress, ↓ scar formation	[74]
**Glycerosomes, hyalurosomes, gly-hyalurosomes**					
*Citrus limon* var. *pompia* fruits	No/No	Primary mouse embryonic fibroblasts (3T3), HaCaT	No	Prevention of oxidative damage; ↑ viability; ↑ biological activity by incorporation of the extract in vesicles, especially gly-hyalurosomes	[26]
**Glycerosomes, liposomes, gluglycerosomes, gel-gluglycerosomes, hyal-glulycerosomes**					
*Hypericum scruglii* aerial parts extract	Polycarbonate dialysis tubes/No	HaCaT	No	↑ antioxidant activity, ↑cell uptake and wound-healing effects	[45]
**Glycerosomes**					
*Rosmarinus officinalis* leaves extract	No/No	No	No	↑ antioxidant activity by liposomal entrapment, better stability of the extract during storage in comparison to free extract	[51]
**Niosomes**					
*Hypericum perforatum* flowering tops	USP dissolution test apparatus/No	No	Adult Mongrel dogs	↓ inflammatory phase, ↑ early beginning of proliferative phase of wound healing, significant ↓ wound size compared to control and Panthenol^®^ 2% cream	[21]
*Calendula* *officinalis* flowers and leaves extract	No/No	Vero cell line	No	↑ wound-healing and protective effect against oxidative stress of *Calendula officinalis* methanolic extract after entrapment into Tween 60 niosomes	[50]
**Santosomes**					
Phycocianin	No/No	HaCaT, endothelial cells, cell uptake	Female CD-1 mice	Easy internalization of phycocyanin from santosomes by keratinocytes and endothelial cells, protective effect against H_2_O_2_ stress; in vivo studies—wound-healing activity	[60]
**Phytosomes**					
*Moringa oleifera* leaves extract	No/No	NHDF	No	Cytocompatibility; ↑ dose-dependent effect in wound closure of filtered *Moringa oleifera* compared to unfiltered samples and controls	[29]
Sinigrin	No/No	HaCaT	No	At the highest tested concentration, 0.14 mg/mL (42 h), the sinigrin–phytosome complex completely cured the wound, whereas the sinigrin alone displayed only 71% wound healing	[71]
*Aloe vera* dry extract	Dialysis/No	HUVECs	No	↑ protective effects in suppressing MGO-induced endothelial cell cytotoxicity, anti-angiogenic effects, ↓ ROS overproduction and induction of oxidative stress; restorative effect on NO production; ↑ expression of several proangiogenic genes: VEGF-A, bFGF, KDR, Ang II, ↓ expression of anti-angiogenic such as Notch I, TGF-β	[19]
**AuNP-phytosomes and liposomes**					
*Calendula officinalis* flowers extract	No/No	NHDF, Vero cell line	No	↑ Antioxidant and wound-healing activity, ↑ stability compared to free forms of each encapsulated material, plain liposome, phytosome form	[70]
**Ethosomes**					
Curcumin	No/No	No	Male Wistar rats	↑ early stages of wound healing, antibacterial activity similar to SSD cream	[80]
*Achillea millefolium* antenna parts	No/Franz cells, rat skin	No	No	↑ skin penetration compared to conventional gel	[103]
**Ethosomes and PEVs**					
*Fraxinus angustifolia* leaves and bark extract	No/No	HaCaT, cell uptake fluorescent vesicles	Male CD-1 mice	↑ local bioavailability of the leaf phytocomplex, ↑ intracellular antioxidant activity in HaCaT, ↑ wound healing in TPA-mouse model for the simple extract ethanolic solution	[27]
**Invasomes**					
Terpenoids ^a^	No/No	No	No	↑ bioavailability of terpenoid-based drugs, strong selective activity against Gram-positive bacteria.	[28]

Legend: ^a^ thymol, menthol, camphor, and 1,8-cineol. SCE—stratum corneum and epidermis, NB1RGB—normal human neonatal skin fibroblasts, NHEK(F)—normal human epidermal keratinocytes, HaCaT—human keratinocytes, HDF—human dermal fibroblast, HGF—human gingival fibroblast, HUVECs—human umbilical vein endothelial cells, SSD—silver sulfadiazine, A.G.—ammonium glycyrrhizate, ULs—ultra-deformable liposomes, MGO- methylglyoxal, E.O.—essential oil, HE—herbal extract, PC—phytocompound.

**Table 6:** Vesicular nanosystems bearing herbal extracts or phytocompounds incorporated in topical systems for wound healing.

VN	Topical Delivery System	Entrapped HE or PC	Ref.
Niosomes	Gel-sodium carboxymethyl cellulose and hydroxyethylcellulose	*Hypericum perforatum* flowering tops	[21]
Liposomes	Thermosensitive gel	*Angelica sinensis* and *Astragali radix* ratio 1:5	[92]
Liposomes	Membranes—CMC, acetylated arrowroot starch	Bromelain extract	[94]
Ethosomes	Carbopol gel	Curcumin	[80]
Liposomes	Collagen-based films	Usnic acid	[31]
Ethosomes	Gel-carbopol 940, hydroxyethylcellulose	*Achillea millefolium* antenna parts	[103]
Niosomes *	Sodium polyacrylate and carbomer mucoadhesive gel	*Zea mays* cobs and *Clitoria ternatea* petals	[85]

Legend: * for healing of oral cavity wounds; VN—vesicular nanosystem; CMC—carboxymethyl cellulose; HE—herbal extract; PC—phytocompound.

## Data Availability

Not applicable.
